# Two new species of the genus *Pseudidonauton* Hering, 1931 from China (Lepidoptera, Limacodidae)

**DOI:** 10.3897/zookeys.1059.68512

**Published:** 2021-09-14

**Authors:** Jun Wu, Alexey V. Solovyev, Hui-Lin Han

**Affiliations:** 1 School of Forestry, Northeast Forestry University, Harbin, 150040, China; 2 Department of Biology and Chemistry, Ulyanovsk State Pedagogical University, Ulyanovsk, 432071, Russia; 3 Key Laboratory of Sustainable Forest Ecosystem Management, Ministry of Education, Northeast Forestry University, Harbin, 150040, China

**Keywords:** Limacodidae, new species, slug caterpillar moths, South-East Asia, taxonomy

## Abstract

Two new species of the genus *Pseudidonauton* Hering (Lepidoptera, Limacodidae), *P.sinensis***sp. nov.** and *P.puera***sp. nov.**, are described from China. The new species are illustrated with images of the adults and male genitalia, and compared with similar species. Distribution maps of these species and a key to all known species of the genus are provided.

## Introduction

The genus *Pseudidonauton* was erected by Hering (1931), with *P.admirabile* Hering, 1931 as its type species. While erecting this genus, Hering proposed that *Idonautonnigribasis* Hampson, 1905 should also belong to this genus (Hering 1931). Later, Holloway (1986) revised the genus and transferred to it *Thoseabhaga* Swinhoe, 1901 and *Idonautonnigribasis* Hampson, 1905. In 2009, three additional species were described, *P.siamica* Solovyev, 2009, *P.chihpyh* Solovyev, 2009 and *P.vexa* Solovyev, 2009, two of which were subsequently recorded from Vietnam (Solovyev 2009; Solovyev and Witt 2009). The type species *P.admirabile* was the first species to be recorded from China (Cai 1981), but we think this record was based on a misidentification. Currently, the genus consists of six described species ranging from India to Sundaland, including *P.admirabile* Hering, 1931, *P.bhaga* (Swinhoe, 1901), *P.nigribasis* (Hampson, 1905), *P.siamica* Solovyev, 2009, *P.chihpyh* Solovyev, 2009 and *P.vexa* Solovyev, 2009. The moths belonging to this genus are small-sized. The antennae in both sexes are filiform, those of the male slightly thickened. The labial palpus is up-curved, with the third segment conspicuous. The proboscis is reduced. The hind tibia has two pairs of spurs. The forewing has R_2–5_ stalked and R_2_ separated behind R_5_; the hindwing has Rs and M_1_ arising from the same place at the upper angle of the cell. The ground colour of forewing is pale brown, and their unique feature is that the basal third of the forewing and the apical area are usually brown. The male genitalia are strongly modified and highly diagnostic for the genus: the uncus is usually broad, flattened, single or bifid; the gnathos is reduced to a small band; the transtilla is well developed and strongly sclerotized, having a large, strongly sclerotized, medial plate that is bifid apically; the valva is generically divided into an upper and a lower part, the lower part having a long spine.

Holloway (1986) recorded the morphology of the larva, pupa and cocoon of *P.nigribasis*, but we have not yet found immature stages of this genus. In this study, we describe two new species, *P.sinensis* sp. nov. and *P.puera* sp. nov., from China, one of which also occurs in Vietnam.

## Material and methods

The specimens were collected with a 220V/450W mercury vapour lamp and a DC black light in southern China and Vietnam. Standard methods for dissection and preparation of the genitalia slides were used (Kononenko and Han 2007). The specimens were photographed using a Nikon D700 camera, whereas the genitalia slides were photographed with an Olympus photo microscope aided by the Helicon Focus software and further processed in Adobe Photoshop CS6. Almost all the type material of the new species is deposited in the collection of Northeast Forestry University (**NEFU**), Harbin, China, except for one male paratype deposited in the collection of Alexey V. Solovyev, Ulyanovsk, Russia (**CASU**). Material from Museum Witt München / Zoologische Staatssammlung München, Munich, Germany (**MWM/ZSM**) was also examined in this study.

## Taxonomic account

### Genus Pseudidonauton

Taxon classificationAnimaliaLepidopteraLimacodidae

Hering, 1931

40FC685C-B17B-5CC2-AE14-ACF1C01BDFF3

Pseudidonauton Hering, 1931, 670, 705. Type species (original designation): Pseudidonautonadmirabile Hering, 1931 [Malay Peninsula: Padang Rengas].

### Pseudidonauton sinensis sp. nov.

Taxon classificationAnimaliaLepidopteraLimacodidae

B21A6E49-9FC1-52F2-89AA-336EC9771C09

http://zoobank.org/47290A63-4817-4513-BC9E-F158A18D3445

**Figures 1–9.**
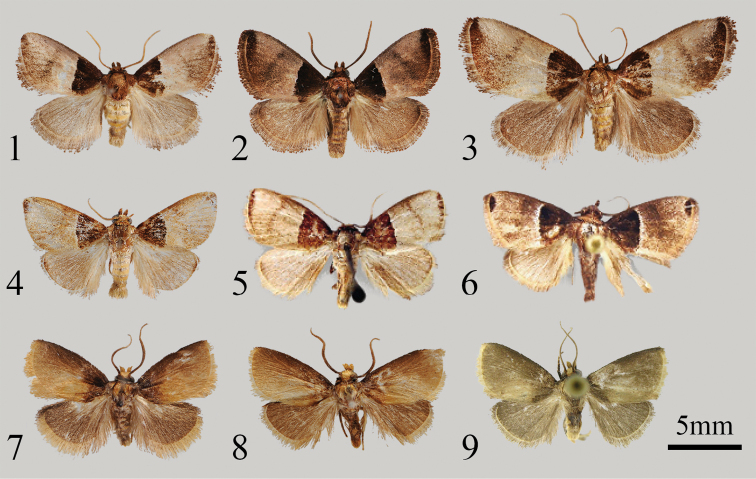
Adults of *Pseudidonauton* spp. **1***P.sinensis* sp. nov., male, holotype **2** ditto, male, paratype **3** ditto, female, paratype **4***P.bhaga* Swinhoe, 1901, male, Borneo, Malaysia (in NEFU) **5***P.chihpyh* Solovyev, 2009, male, holotype (in MWM/ZSM) **6***P.vexa* Solovyev, 2009, male, holotype (in MWM/ZSM) **7***P.puera* sp. nov., male, holotype **8** ditto, male, paratype **9** ditto, male, paratype (in CASU). Scale bar: 5 mm.

**Figures 10–19.**
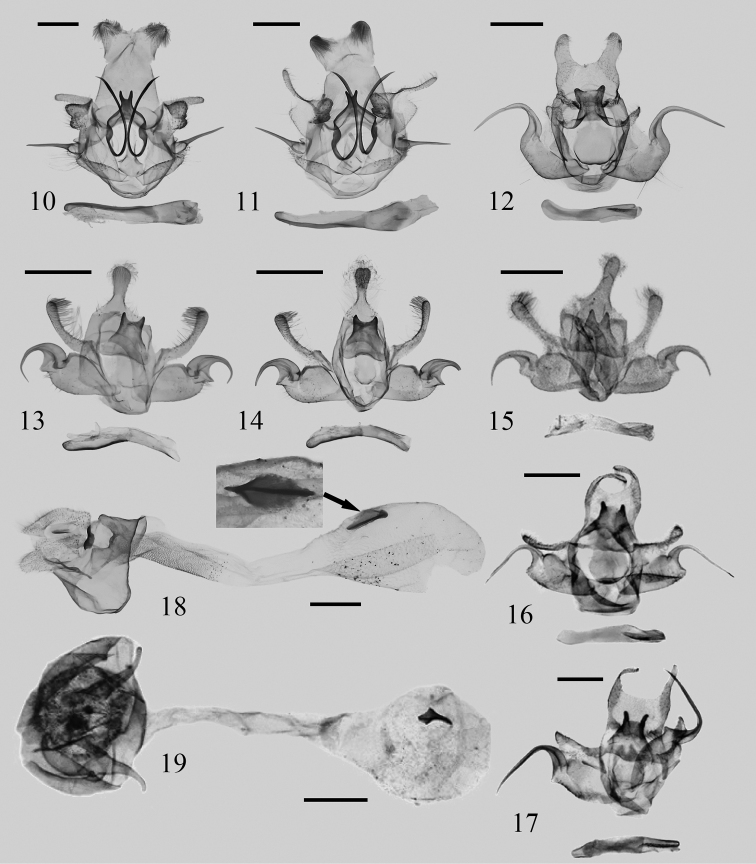
Genitalia of *Pseudidonauton* spp. **10***P.sinensis* sp. nov., male, holotype **11** ditto, male, paratype **12***P.bhaga* Swinhoe, 1901, male, Borneo, Malaysia, genitalia No. WuJ-392–1 (in NEFU) **13***P.puera* sp. nov., male, holotype **14** ditto, male, paratype **15** ditto, male, paratype (in CASU) **16***P.chihpyh* Solovyev, 2009, male, holotype (in MWM/ZSM) **17***P.vexa* Solovyev, 2009, male, holotype (in MWM/ZSM) **18***P.sinensis* sp. nov., female, paratype; **19***P.vexa* Solovyev, 2009, female, paratype (in MWM/ZSM). Scale bars: 0.5 mm.

Figures 1 , 2 , 3 , 10 , 11 , 18

#### Holotype.

♂, China, Chongqing Municipality, Mt. Simian, 29.VII–2.VIII.2020, leg. HL. Han and J. Wu, genit. prep. WuJ-388–1 (NEFU).

#### Paratypes.

15♂, 1♀, same data as for holotype, genit. prep. for four dissected paratypes WuJ-288–1, 289–1, 387–1 and 389–2 (NEFU); 3♂, 1♀, China, Prov. Guizhou, Zunyi City, Shierbeihou scenic spots, Shuanghe village, 3–5.VIII.2020, leg. HL. Han and J. Wu, genit. prep. for three dissected paratypes WuJ-382–1, 383–1 and 384–2 (NEFU); 4♂, China, Prov. Guizhou, Zunyi City, Xishui County, Sanchahe Town, 1.VII.2019, leg. MR. Xing, BX. Zhao, and H. Sun (NEFU); 4♂, China, Prov. Jiangxi, Guanshan Nature Reserve, 21–27.VIII.2017, leg. GX. Wang and WJ. Li, genit. prep. for two dissected paratypes WuJ-342–1, 343–1 (NEFU); 1♂, China, Prov. Jiangxi, Guanshan Nature Reserve, 21–23.VIII.2017, leg. HL. Han, genit. prep. WuJ-379–1 (NEFU); 41♂, China, Prov. Zhejiang, Pan’an County, Mt. Dapan, 25.VI–6.VII.2019, leg. J. Wu and JJ. Fan, genit. prep. for four dissected paratypes WuJ-390–1, 440–1, 441–1, 442–1 (NEFU); 3♂, China, Prov. Zhejiang, Jiangshan City, Laofoyan Village, 3.VII.2017, leg. ZG. Zhang, YY. Jia and J. Li (NEFU); 1♂, China, Prov. Zhejiang, Jiangshan City, Xiayangping Village, 4.VII.2017, leg. ZG. Zhang, YY. Jia and J. Li (NEFU); 2♂, China, Prov. Fujian, Mt. Wuyi, Taohuayu, 6.VIII.2020, leg. MJ. Qi and XY. Jin (NEFU); 3♂, China, Aut. Reg. Xizang, Linzhi City, Lulang station, 15.VII.2017, leg. HL. Han, genit. prep. for three dissected paratypes WuJ-379–1, 380–1, 381–1 (NEFU).

#### Diagnosis.

The new species is very similar to its congeners in appearance, especially to *P.bhaga* (Fig. 4), *P.chihpyh* (Fig. 5) and *P.vexa* (Fig. 6), but it can be distinguished from these species by having no distinct borderline between the apical patch and the ground colour in the forewing. Moreover, the whole outer margin area in the new species is covered by a conspicuous dark brown smudge.

The male genitalia are clearly different from those of the other congeners: in *P.sinensis* sp. nov. (Figs 10, 11), the uncus is shallowly divided into two parts; the transtilla is narrow apically and bearing a pair of long slender spine-like process at the base; the saccular process is straight. The vesica lacks cornuti. However, in *P.bhaga* (Fig. 12), *P.chihpyh* (Fig. 16) and *P.vexa* (Fig. 17), the uncus is deeply divided into two parts; the apical plate of the transtilla is broad, without a slender spine-like process at the base; the sacculus process is strongly curved. The vesica bears a row of small cornuti.

In the female genitalia, the diagnostic difference between *P.sinensis* sp. nov. (Fig. 18) and *P.vexa* (Fig. 19) is that the former has a thick ductus bursae, and the surface of the 1/2 near the ostium bursae is rough; a leaf-shaped signum is located at the upper part of the corpus bursae.

#### Description.

Adult (Figs 1, 2, 3). Wingspan 14–16 mm in male, 18–20 mm in female. Head brown; labial palpus up-curved; antenna filiform in both sexes, brown. Thorax and tegula brown. Scales on legs brown mixed with a little yellow. Forewing ground colour pale brown to reddish brown, basal 1/3 with unique dark brown area. Apical patch dark brown, no visible borderline with the ground colour. Whole outer margin area covered by a conspicuous dark brown smudge. Median line slightly visible, arched, dark brown, runs from costal at ca. 3/5 distance from wing base to tornus. In some individuals, median line barely visible. Fringe long, brown. Hindwing ground colour slightly darker than forewing, reddish brown; apex dark brown; fringe long, brown. On abdomen, hair covering each abdominal segment golden yellow mixed with pale brown, with long pale brown hairs in terminal area.

**Male genitalia** (Figs 10, 11). Uncus broad, flattened, weakly divided into two parts, each covered with dense hairs on surface. Gnathos reduced. Tegumen broad, slightly trapezoidal. Transtilla well developed and strongly sclerotized, with small, apically bifid medial plate; basal part of transtilla bearing pair of long, slender, spine-like process slightly enlarged at base. Valva strongly modified and clearly divided into upper and lower parts: upper part finger-shaped, with ear-like process at base and nearly membranous triangular structure behind it; in lower part, sacculus slightly inflated, with swollen base and straight spine-like sacculus process ca. 1/2 length of phallus. Juxta flattened, slightly concave in middle of apex. Saccus not obvious. Phallus slender, tube-shaped, slightly sclerotized terminally; vesica without cornuti.

**Female genitalia** (Fig. 18). Ovipositor lobes ear-shaped, covered with dense hairs on surface. Postvaginal plate strongly sclerotized. Apophysis anterioris very short, with only a small spine; apophysis posterioris long, ca. 2/3 length of ovipositor lobes, inflated at base and blunt at apex. There is a distinct, nearly square incision on the ostium bursae. Ductus bursae thick, not spiral-shaped, with rough sclerotized surface on upper half, membranous on lower half. Corpus bursae pear-shaped, with tiny hairs on surface and a strongly sclerotized, leaf-shaped signum on upper 1/3.

#### Distribution.

**Figure 20.**
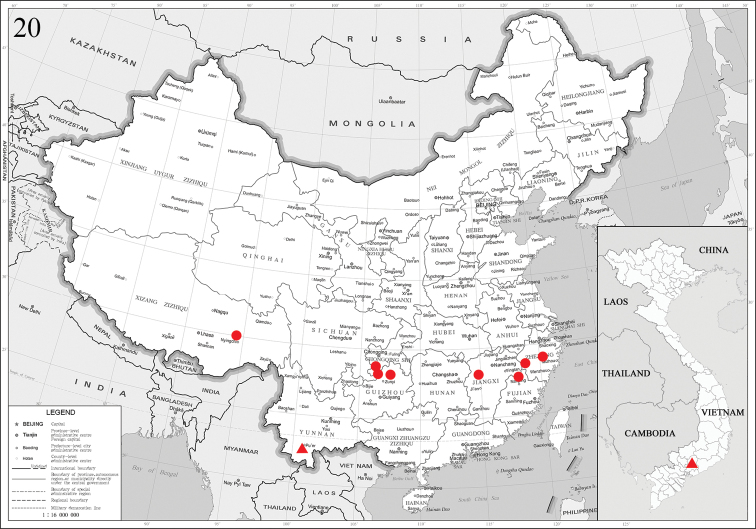
Distribution map of two new species of *Pseudidonauton*: circle: *P.sinensis* sp. nov. (China: Chongqing, Zhejiang, Guizhou, Fujian, Jiangxi, Xizang); triangle: *P.puera* sp. nov. (China: Yunnan; Vietnam: Dong Nai).

China (Chongqing, Zhejiang, Guizhou, Fujian, Jiangxi, Xizang) (Fig. 20).

#### Etymology.

The species is named *sinensis* because of its wide distribution in China.

#### Bionomics.

The specimens were collected from June **t**o August at altitudes of 560–2,800 m.

### Pseudidonauton puera sp. nov.

Taxon classificationAnimaliaLepidopteraLimacodidae

B671B741-CB80-52EA-8C8C-6300F2010680

http://zoobank.org/31B3CA5B-866B-4240-A92A-F7CEA1EC4B1C

Figures 7 , 8 , 9 , 13 , 14 , 15

#### Holotype.

♂, China, Prov. Yunnan, Puer City, Manxieba, 3.VIII.2018, leg. HL. Han, J. Wu, MR. Li, genit. prep. WuJ-237–1 (NEFU).

#### Paratypes.

1♂, same data as for holotype, genit. prep. WuJ-236–1 (NEFU); 1♂, Vietnam, Dong Nai, Vinh Cuu Nat. Res., Phu Ly, Dakinde, 11.41203°N, 107.10508°E, 106 m a.s.l., 27.VI.2011, leg. A. Solovyev, S. Pugaev, S. Nedoshivina, genit. prep. 0234 (CASU).

#### Diagnosis.

The new species can be clearly distinguished from the known species in its appearance: the dark brown area at the base of the forewing has no obvious borderline with the ground colour and has no apical patch. In the male genitalia, the new species is similar to *P.chihpyh* (Fig. 16), but uncus rod-shaped and not divided into two parts apically is the main combination that distinguishes the new species from *P.chihpyh* and from all other known species in this genus.

#### Description.

Adult (Figs 7, 8, 9). Wingspan 14 mm in male. Head brown, with golden scales on the frons; labial palpus up-curved, golden; antenna filiform in male, brown. Thorax golden, mixed brown scales; tegula brown. Scales on legs dark brown to golden. Forewing ground colour yellowish brown, dark brown at ca. 1/3 from wing base and costal margin area and no clear borderline with the ground colour; fringe long, especially in the tornus area, yellow; forewing without other lines. Hindwing ground colour reddish brown; fringe long, yellow to light brown, but dark brown in costal area. Abdomen dark brown, with light brown and golden hairs between each abdominal segment.

**Male genitalia** (Figs 13, 14, 15). Uncus short, rod-shaped, slightly enlarged and rounded apically, covered with dense hairs. Gnathos reduced to a small plate. Tegumen broad. Transtilla very developed and strongly sclerotized, wide at base, slightly narrower in apical part and with semicircular depression in middle of apex. Valva strongly modified, clearly divided into two parts: upper part finger-shaped, slightly thin at middle and covered with dense hairs on apex; in basal part, sacculus visibly enlarged, bearing swollen base and eagle-claw-shaped sacculus process; small triangular structure located at base of sacculus process. Juxta shield-shaped. Saccus not obvious. Phallus slender, tube-shaped, slightly curved at middle.

**Female genitalia.** Unknown.

#### Distribution.

China (Yunnan), Vietnam (Dong Nai) (Fig. 20).

#### Etymology.

The species is named *puera* (a noun in apposition) after its type locality in Puer City, Prov. Yunnan, China, which is famous for Puer tea.

#### Bionomics.

The moths fly in late June and August. The Chinese specimens were collected with a light trap close to a coniferous forest; the main vegetation around the collecting site consists of *Pinusyunnanensis* Franch. (Pinaceae).

### Key to the species of *Pseudidonauton* based on male genitalia, with distributions

**Table:** 

1	Uncus not bifid	**2**
–	Uncus divided into two parts (bifid)	**3**
2	Uncus not rod-shaped; medial process of transtilla fish-tail-like; basal part of valva with large pads; saccular spine long	***P.siamica* Solovyev (northern Thailand, central Vietnam)**
–	Uncus rod-shaped; medial process of transtilla broad and not fish-tail-like; basal part of valva without pads; saccular spine short	***P.puera* sp. nov. (south-western China, south-eastern Vietnam)**
3	Uncus with a V-shaped cleft	***P.admirabile* Hering (Peninsular Malaysia, ? southern China)**
–	Uncus with a U-shaped cleft	**4**
4	Uncus bearing long, strong spurs; upper part of valva longer than lower part	***P.nigribasis* (Hampson) (India)**
–	Uncus without spurs; upper part of valva shorter than lower part	**5**
5	Medial notch of uncus narrow, lateral parts of uncus as wide as 1/3 of uncus wdth	**6**
–	Medial notch of uncus broad, lateral parts of uncus as wide as 1/4 of uncus wdth	**7**
6	Medial notch of uncus shallow, basal part of transtilla bearing pair of long, slender, spine-like processes	***P.sinensis* sp. nov. (southern China)**
–	Medial notch of uncus deep, basal part of transtilla without processes	***P.bhaga* (Swinhoe) (Borneo, Peninsular Malaysia, Sumatra)**
7	Small papula-shaped acute process present near saccular spine	***P.vexa* Solovyev (northern and central Vietnam, south-eastern Thailand)**
-	Papula-shaped process absent near saccular spine	***P.chihpyh* Solovyev (China: Taiwan)**

## Discussion

The genus *Pseudidonauton* comprises eight species in total to date, but the presence of *P.admirabile* in China is still unclear. It is possible that the record from southern China of this species by Cai (1981) is based on a misidentification. Cai described the uncus of two male specimens to have a U-shaped cleft, but the same structure is V-shaped in the holotype of *P.admirabile* (Solovyev 2009). In the paper of Cai, only a blurred picture of an adult was given, but no images of the genitalia were provided. As we did not examine these two old specimens (collected in 1956), we cannot make a final decision on whether this is a misidentification of *P.sinensis* sp. nov., and more specimens are needed for comparison.

In addition, two unidentified females of *Pseudidonauton* were collected from southern China (Guangdong and Hainan Province) in 2017 and 2019, respectively; the female from Hainan may belong to a species known from Vietnam. However, due to the lack of male specimens at present, we cannot accurately identify which species they belong to.

## Supplementary Material

XML Treatment for Pseudidonauton

XML Treatment for Pseudidonauton sinensis

XML Treatment for Pseudidonauton puera
